# Pathways to Improving Mental Health in Compassion-Focused Therapy: Self-Reassurance, Self-Criticism and Affect as Mediators of Change

**DOI:** 10.3389/fpsyg.2018.02442

**Published:** 2018-12-05

**Authors:** Marion Sommers-Spijkerman, Hester Trompetter, Karlein Schreurs, Ernst Bohlmeijer

**Affiliations:** ^1^Centre for eHealth and Well-being Research, Department of Psychology, Health and Technology, University of Twente, Enschede, Netherlands; ^2^Center of Research on Psychological and Somatic Disorders, Department of Medical and Clinical Psychology, Tilburg University, Tilburg, Netherlands; ^3^Roessingh Research and Development, Enschede, Netherlands; ^4^Optentia Research Focus Area, North-West University (VTC), Vanderbijlpark, South Africa

**Keywords:** compassion-focused therapy, mediators, self-reassurance, self-criticism, affect

## Abstract

**Objective:** The working mechanisms of compassion-focused therapy (CFT) remain understudied. Drawing on the theoretical model underlying CFT, we examined four putative working mechanisms – self-reassurance, self-criticism, positive/negative affect – in relation to changes in well-being and psychological distress.

**Methods:** Data of a waitlist randomised controlled trial (*N* = 242) investigating the effectiveness of a self-help CFT-intervention in a non-clinical sample were analysed. Using single and multiple mediation models, we assessed if changes in self-reassurance, self-criticism and positive/negative affect during the intervention (3-month interval) mediated changes in well-being and depressive/anxiety symptoms from baseline to follow-up (6-month interval) compared to the waitlist condition.

**Results:** For each outcome, single analyses revealed that the effects of CFT were significantly mediated by self-reassurance and self-criticism. The mediating role of affect differed across outcomes. In combined models, self-reassurance emerged as a significant mediator for well-being and anxiety symptoms. Additionally, positive and negative affect were found significant mediators of the effects on depressive and anxiety symptoms, respectively.

**Conclusion:** This study provides preliminary empirical evidence that CFT operates through cultivating self-reassurance, reducing self-criticism and regulating positive and negative affect in a non-clinical sample. To advance the development of CFT, further exploration of therapeutic change processes and their interplay is needed.

## Introduction

Over the past decade, a growing body of empirical evidence has accumulated that testifies to the effectiveness of compassion-based interventions as a means of promoting mental health (Leaviss and Uttley, 2015; Kirby, 2016; Kirby et al., 2017b). The most abundant evidence has come from studies investigating compassion-focused therapy (CFT), showing favourable effects over a wide range of well-being and distress outcomes (e.g., Gilbert and Procter, 2006; Shapira and Mongrain, 2010; Braehler et al., 2013). Yet what remains unclear is how and why CFT works. This is not specific to CFT, but is true for many clinical interventions (Kraemer et al., 2002; Kazdin, 2009).

Given the growing interest for CFT and other compassion-based interventions and a general renewed interest in change processes leading to psychotherapeutic change (Laurenceau et al., 2007; Elliot, 2010), we believe exploring mediators and mechanisms underlying the effectiveness of CFT is a timely and fruitful area for investigation. Not only will this strengthen the existing theoretical framework of CFT, but also will it guide the development and refinement of CFT-based interventions, hence advance clinical practise and research in this burgeoning field.

Compassion-focused therapy strives for a compassionate mind which includes the ability to be compassionate toward the self and others as well as to receive compassion from others. Compassion is viewed as a multidimensional construct encompassing two interrelated mindsets (Gilbert, 2014). The first mindset involves the ability to be sensitive to the suffering of self and others, relating to a multitude of attributes, such as the motivation to care and the capacity for feeling sympathy and empathy. The second mindset, commitment to alleviating suffering, requires a particular set of affiliative skills in the sphere of attention, cognition, behaviour and emotion conducive to the development of a compassionate mind. Examples include the ability to replace self-critical thoughts with compassionate self-correction and to generate compassionate feelings for the self and others. Besides psycho-education on the human mind, CFT employs various techniques designed to build a compassionate mind, such as mindfulness techniques, compassionate imagery, expressive writing, practising soothing breathing rhythm, and practising compassionate ways of thinking (Gilbert, 2014).

Drawing on the evolutionary-based theoretical model underlying CFT, several mechanisms of change can be identified: (1) cultivating self-reassurance; (2) disengaging from self-critical thoughts; (3) stimulating attention for and processing of positive affect; and (4) improving distress tolerance and decreasing negative affect (Gilbert, 2014). Cultivating self-reassurance can be seen as the primary and central presumed mechanism of CFT, whereas the other three are secondary mechanisms that evolve from the development of self-reassurance. Together, these change processes are thought to facilitate increases in well-being and decreases in psychological distress.

Cultivating self-reassurance as the central mechanism of CFT reflects the ability to relate to the self in a warm, soothing and reassuring manner when encountering setbacks or failures (Gilbert et al., 2004). This is deemed a core facet of compassion toward the self. Matos et al. (2017a) showed that self-reassurance is positively associated with the ability to embody one’s compassionate self in everyday life. Other research has shown that practising CFT has potential to improve self-reassurance (Gilbert and Procter, 2006; Lucre and Corten, 2013; Sommers-Spijkerman et al., 2018c), which, in turn, positively relates to well-being and negatively relates to psychopathology including stress, depressive and anxiety symptoms (Gilbert et al., 2008; Castilho et al., 2015; Sommers-Spijkerman et al., 2018b).

The CFT model presumes that the development of self-reassurance – among others – facilitates the ability to reduce self-critical thinking (Gilbert, 2014). Consistent with this notion, a recent study demonstrated that self-reassurance protects against the depressogenic effect of self-criticism (Petrocchi et al., 2018). Whereas previous research has shown that CFT leads to significant reductions in self-criticism (e.g., Gilbert and Procter, 2006; Matos et al., 2017b), to our knowledge, no studies have assessed whether the beneficial effects of CFT on well-being and distress can be attributed to improvements in self-criticism.

The third and fourth mechanisms serve to affect regulation of both positive and negative emotions, another important treatment target of CFT. In CFT, compassion is related to an evolutionary functional analysis of emotions, distinguishing between three major affect regulation systems: (1) the threat protection system, which provides abilities to detect and respond to threat; (2) the drive and resource-seeking system, which provides information on the availability of resources and rewards; and (3) the soothing and affiliation system, which enables individuals to reassure and soothe themselves (Gilbert, 2009, 2014). CFT is thought to strengthen the capacity for experiencing and tolerating soothing emotions in the face of setbacks, thereby fostering positive affective states such as safeness, calmness and contentment, while alleviating negative affective states through enabling people to regulate and engage with unpleasant or feared emotions characteristic for the threat system, including anger, anxiety and guilt (Gilbert, 2009, 2014). Consistently, a number of experimental studies have corroborated the notion that practising compassion up-regulates positive affect (Engen and Singer, 2015) and down-regulates negative affect (Leary et al., 2007; Diedrich et al., 2014, 2016; Arimitsu and Hofmann, 2017). In turn, positive affect has been positively associated with well-being and negatively associated with psychopathology in numerous studies, and vice versa for negative emotions (Lyubomirsky et al., 2005; Fredrickson et al., 2008; Cohn et al., 2009; Lyubomirsky and Layous, 2013; Seaton and Beaumont, 2015).

Thus far, direct empirical evidence to support the mechanisms of change put forward in the theoretical framework of CFT is lacking, although existing randomised controlled trials (RCTs) offer ample opportunity for an initial exploration of potential mediators of CFT-induced changes in mental health. A recently conducted waitlist RCT showed that CFT as guided self-help elicits favourable effects on (among others) well-being, depression, anxiety, self-compassion, self-reassurance, self-criticism and affect in an adult community sample with low to moderate levels of well-being and mild distress (Sommers-Spijkerman et al., 2018a). Building on these findings, the aim of the current study was to examine the unique and combined indirect effects of the four putative working mechanisms or mediators outlined in the CFT model, namely self-reassurance, self-criticism and positive/negative affect, in changing levels of well-being and psychological distress in CFT, compared to a waitlist control condition. For well-being as well as depressive and anxiety symptoms, we examined the extent to which the four putative mediators independently and together mediated the effects of CFT over a 6-month period. First, we tested the hypothesis that changes in self-reassurance, self-criticism, and positive and negative affect during the intervention significantly mediated the effects of CFT on well-being and depressive/anxiety symptoms, compared to the waitlist condition. Given the focus and contents of the CFT intervention, a second hypothesis was that changes in both levels of well-being and levels of depressive/anxiety symptoms at 6 months would be predominantly mediated by changes in self-reassurance during the intervention. Nonetheless, it was postulated that all mediators would play a role in the effectiveness of CFT regardless of outcome.

## Materials and Methods

### Participants and Procedure

The current study builds further upon the findings of a recently conducted RCT (Sommers-Spijkerman et al., 2018a). The RCT was approved by the Faculty of Behavioral Sciences Ethics Committee at the University of Twente (BCE15354) and registered in the Netherlands Trial Register (NTR5413).

The RCT sample consisted of adults with low to moderate levels of well-being recruited through advertisements in Dutch national newspapers. Eligibility criteria were: (1) an age of 18 years or older; (2) low to moderate levels of well-being according to the Mental Health Continuum-Short Form (MHC-SF; Lamers et al., 2011); (3) access to a computer or tablet with a good internet connection; (4) possession of an e-mail address; (5) sufficient proficiency in the Dutch language (reading and writing); and (6) (online) informed consent. Applicants who were experiencing a high level of well-being (i.e., prior to flourishing on the MHC-SF) (Lamers et al., 2011) or who had a score > 11 on the depression and/or anxiety subscale of the Hospital Anxiety and Depression Scale on initial screening (HADS; Zigmond and Snaith, 1983) were excluded.

**FIGURE 1:**
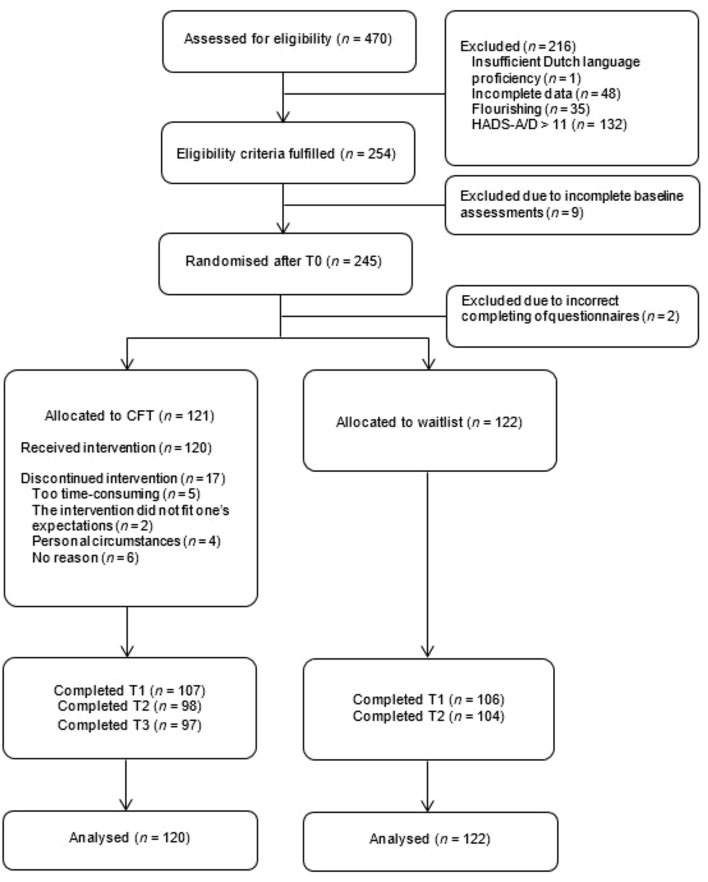
Flowchart of study participants and dropouts.

Of the 470 applicants assessed for eligibility, 243 participants were randomly allocated to either CFT (*n* = 121) or a waitlist control condition (*n* = 122). One participant in the CFT group withdrew from the study prior to the start of the intervention and was therefore not included in the analyses. The flow of participants through the study and baseline characteristics can be found in Figure 1 and Table 1, respectively. The conditions did not significantly differ on any of the demographic variables, process measures or outcomes at baseline (*p* ≥ 0.06).

**Table 1:** Baseline characteristics of the participants (*N* = 242).

	Total	CFT	WLC
	(*n* = 242)	(*n* = 120)	(*n* = 122)
**Age, years**			
Mean (*SD*)	52.87 (9.99)	52.83 (9.78)	52.90 (10.22)
Range	20–78	20–78	26–78
**Gender, *n* (%)**			
Male	61 (25.2)	24 (20.0)	37 (30.3)
Female	181 (74.8)	96 (80.0)	85 (69.7)
**Nationality, *n* (%)**			
Dutch	242 (100.0)	120 (100.0)	122 (100.0)
Other	–	–	–
**Marital status, *n* (%)**			
Married/registered partnership	131 (54.1)	62 (51.7)	69 (56.6)
Not married (never married, divorced, widowed)	111 (45.9)	58 (48.3)	53 (43.4)
**Living situation, *n* (%)**			
With partner	159 (65.7)	76 (63.3)	83 (68.0)
Without partner	83 (34.3)	44 (36.7)	39 (32.0)
**Education level (highest level completed), *n* (%)**			
Low (primary school, lower vocational education)	1 (0.4)	–	1 (0.8)
Intermediate (secondary school, vocational education)	28 (11.6)	17 (14.2)	11 (9.0)
High (higher vocational education, university)	213 (88.0)	103 (85.8)	110 (90.2)
**Daily activities, *n* (%)**			
Paid employment	184 (76.0)	92 (76.7)	92 (75.4)
No paid employment	53 (21.9)	25 (20.8)	28 (23.0)
Student	5 (2.1)	3 (2.5)	2 (1.6)


*CFT, compassion-focused therapy; WLC, waitlist control group.*

### Intervention

The CFT-intervention consisted of a self-help book entitled “Compassion as key to happiness” (Hulsbergen and Bohlmeijer, 2015) that could be worked through in 7 to 9 weeks, with weekly email support from a trained counsellor. In seven lessons based on the CFT approach (Gilbert, 2014), psycho-education regarding compassion, self-reflective and experiential exercises and fictional narratives were used to cultivate compassionate attributes and skills. Downloadable audio exercises were part of the first four lessons. Although the intervention was self-administered, e-mail guidance was offered for personal support, positive reinforcement and increasing adherence. A more extensive description of the intervention can be found in Sommers-Spijkerman et al. (2018b).

Participants in the waitlist control group were not offered any intervention, but were free to access any other form of care. Six months after baseline, these participants were offered the CFT self-help book without email counselling.

### Measures

In the present study, we used assessments from the RCT performed at baseline (T0), post-intervention at 3 months (T1), and 3-month follow-up (T2; i.e., 6 months after baseline).

#### Outcome Variables

Outcome measures were the Mental Health Continuum-Short Form (MHC-SF) and the Hospital Anxiety and Depression Scale (HADS). In this study, the baseline and 3-month follow-up data were used.

The MHC-SF (14 items, score 0–5) measures well-being in three dimensions: (1) emotional well-being (3 items), defined in terms of experiencing positive emotions and life satisfaction; (2) psychological well-being (6 items), defined in terms of positive individual functioning; and (3) social well-being (5 items), defined in terms of positive functioning in society. Participants rated their well-being in the past month, with higher scores reflecting a higher level of well-being. The MHC-SF has been shown to be a valid and reliable instrument (Lamers et al., 2011). In the present study, internal consistency of the total scale was good for all measurements, with α ≥ 0.84.

The HADS was used to assess two indicators of psychological distress, i.e., depressive symptoms (HADS-D, 7 items, score 0–21) and anxiety symptoms (HADS-A, 7 items, score 0–21), in the previous week (Zigmond and Snaith, 1983). Higher scores indicate more depressive or anxiety symptoms. Previous research indicates satisfactory psychometric properties for the HADS (Spinhoven et al., 1997; Bjelland et al., 2002; Stern, 2014). In the current study, internal consistency of both subscales was deemed acceptable for each assessment (HADS-D: α ≥ 0.72; HADS-A: α ≥ 0.69).

#### Mediators

The baseline and post-intervention data of the following two process outcome measures were used: the Forms of Self-Criticising/Attacking and Self-Reassuring Scale (FSCRS) and the Positive and Negative Affect Schedule (PANAS).

The FSCRS comprises three subscales. The first subscale called “reassured self” measures feelings of soothing and reassurance (8 items, score 0–32). The second and third subscale assess self-criticism by measuring both feelings of inadequacy and inferiority (i.e., inadequate self, 9 items, score 0–36) and feelings of self-hatred and self-contempt (i.e., hated self, 5 items, score 0–20). In the present study, the reassured self subscale and inadequate self subscale were used to assess self-reassurance and self-criticism, respectively. Higher scores mean a higher level of self-reassurance or self-criticism. The hated self subscale was not used for several reasons. First, this subscale represents a more pathological form of self-criticism which is deemed less relevant in a non-clinical sample. Consistently, previous findings from the RCT (Sommers-Spijkerman et al., 2018a) have shown a ceiling effect for hated self with low baseline scores leaving little room for improvement. Second, the CFT intervention evaluated in the RCT is not so much targeted at hated self but rather at inadequate self. Multiple studies have indicated that the FSCRS has good reliability and construct validity (Gilbert et al., 2004; Kupeli et al., 2013; Baião et al., 2015; Castilho et al., 2015). In the current study, internal consistency was satisfactory with alphas ≥ 0.79 for self-reassurance and alphas ≥ 0.83 for self-criticism.

The PANAS (20 items) examines positive affect (10 items, score 10–50) and negative affect (10 items, score 10–50), with higher scores reflecting greater levels of positive and negative affect, respectively. The PANAS has shown adequate internal consistency, test-retest reliability and construct validity (PANAS; Watson et al., 1988; Peeters et al., 1996; Boon and Peeters, 1999). In this study, we found alphas ≥ 0.85 and ≥ 0.80 for positive and negative affect, respectively.

### Summary of RCT Findings

As reported in Sommers-Spijkerman et al. (2018a), analyses using repeated measures analysis of variance (ANOVA) indicated superior improvement in the CFT group relative to the waitlist control group at both 3 and 6 months on all outcome and process variables, except for positive affect for which superior improvement was only observed at post-intervention. Similar effect sizes were observed at post-intervention (*d*: 0.28 to 0.59) and 3-month follow-up (*d*: 29 to 0.56). No significant moderators were found. Adherence was high; CFT participants completed on average 6.6 lessons (*SD* = 1.0) and 74% (*n* = 89) worked through all seven lessons. Taken together, the results suggest that CFT as bibliotherapy intervention with email support has both short-term and long-term beneficial effects on mental health.

### Analyses

Statistical analyses were in agreement with the intention-to-treat (ITT) principle. Missing data were imputed by applying the expectation–maximisation (EM) algorithm (Dempster et al., 1977; El-Masri and Fox-Wasylyshyn, 2005). Available data was 100, 88 and 83.5% at T0, T1, and T2, respectively. Before testing the mediation models, the interrelationships between the mediation variables (i.e., T0-T1 change scores on self-reassurance, self-criticism, positive affect and negative affect) and outcome variables (i.e., T0-T2 change scores on well-being and depressive/anxiety symptoms) were examined using Pearson’s correlation coefficient (two-tailed). Correlations < 0.10 were considered weak, correlations between 0.10 and 0.30 were considered small, correlations between 0.30 and 0.50 were considered moderate and correlations between 0.50 and 1.00 were considered strong (Cohen, 1988). Mediational analyses were conducted using the PROCESS macro developed by Hayes (2012) in SPSS 23.0, following the regression-based path analysis framework of Preacher and Hayes (2004, 2008). First, the cross-product of the coefficient for the relationship between condition (X; CFT = 1, WLC = 0) and mediator (M) (a-path) was calculated. Then, the cross-product of the coefficient for the relationship between the mediator (M) and outcome variable (Y) while controlling for X (b-path) was calculated. Finally, the overall significance of the a^∗^b effect was tested. For each outcome measure (i.e., well-being, depressive symptoms and anxiety symptoms), we assessed whether changes from T0 to T2 in CFT compared to the waitlist control condition were indirectly affected by changes in self-reassurance, self-criticism and positive/negative affect from T0 to T1, correcting for baseline scores on both M and Y. Both simple and multiple mediation models were tested, wherein indirect effects (ab) as well as their corresponding bias-corrected 95% confidence intervals (CIs) were generated. All analyses were based on 5000 bootstrap samples. When the CI did not include zero, the indirect effect was considered significant. A simple mediation model was tested per outcome, wherein the T0-T2 change score on the respective outcome measure was entered as the dependent variable (Y), the dummy variable representing condition (CFT = 1, WLC = 0) was entered as the independent variable (X), the T0-T1 change score on self-reassurance, self-criticism, positive affect or negative affect was entered as the mediator (M), and the baseline scores on Y and M were entered as covariates. For each outcome measure, multiple mediation models were also tested by simultaneously entering all putative mediators in the model.

## Results

### (Inter)Correlations Between Mediators and Outcomes

Means and *SD*s for all outcomes and mediators are presented in Table 2. T0-T1 changes in self-reassurance, self-criticism and positive/negative affect were found to be significantly correlated with one another (see Table 3). A strong correlation was observed between changes in self-reassurance and changes in positive affect. All remaining correlations were either moderate or small. T0-T2 changes in well-being showed a strong correlation with T0-T2 changes in depressive symptoms and a moderate correlation with T0-T2 changes in anxiety symptoms. Furthermore, a strong correlation was found between changes in depressive and anxiety symptoms. All three outcomes showed small correlations with T0-T1 changes in self-reassurance, self-criticism and positive/negative affect.

**Table 2:** Means and *SD*s of mediating and outcome variables.

Measures	Assessment	CFT	WLC
		(*n* = 120)	(*n* = 122)
		Mean (*SD*)	Mean (*SD*)
MHC-SF – well-being	Baseline	2.35 (0.65)	2.48 (0.65)
	Post	2.94 (0.73)	2.57 (0.67)
	3M-FU	3.01 (0.71)	2.74 (0.68)
HADS – depressive symptoms	Baseline	6.39 (3.26)	6.30 (3.06)
	Post	4.17 (3.33)	5.73 (3.42)
	3M-FU	4.05 (3.00)	5.12 (3.45)
HADS – anxiety symptoms	Baseline	8.13 (2.94)	7.97 (2.99)
	Post	6.01 (3.22)	7.26 (3.27)
	3M-FU	5.57 (2.68)	6.86 (3.38)
FSCRS – self-reassurance	Baseline	16.18 (4.99)	16.34 (5.03)
	Post	19.46 (4.75)	17.20 (5.25)
	3M-FU	20.04 (4.80)	17.75 (5.23)
FSCRS – self-criticism	Baseline	18.47 (7.29)	18.46 (6.66)
	Post	14.58 (6.03)	17.19 (6.97)
	3M-FU	12.70 (5.85)	16.45 (7.67)
PANAS – positive affect	Baseline	32.57 (5.80)	31.99 (6.06)
	Post	35.00 (5.86)	32.67 (6.14)
	3M-FU	35.14 (5.12)	33.55 (5.80)
PANAS – negative affect	Baseline	22.35 (6.07)	22.24 (5.69)
	Post	19.12 (5.55)	21.12 (6.06)
	3M-FU	18.55 (4.81)	20.45 (5.87)


*3M-FU, 3-month follow-up; CFT, compassion-focused therapy; FSCRS, Forms of Self-Criticising/Attacking and Self-Reassuring Scale; HADS, Hospital Anxiety and Depression Scale; MHC-SF, Mental Health Continuum–Short Form; PANAS, Positive and Negative Affect Schedule; WLC, waitlist control group.*

**Table 3:** (Inter)correlations between mediating and outcome variables (*N* = 242).

	1	2	3	4	5	6	7
1. Self-reassurance^a^	-						
2. Self-criticism^a^	-0.382^∗∗∗^	-					
3. Positive affect^a^	0.518^∗∗∗^	-0.264^∗∗∗^	-				
4. Negative affect^a^	-0.341^∗∗∗^	0.373^∗∗∗^	-0.379^∗∗∗^	-			
5. Well-being^b^	0.257^∗∗∗^	-0.128^∗^	0.280^∗∗∗^	-0.199^∗∗^	-		
6. Depressive symptoms^b^	-0.227^∗∗∗^	0.185^∗∗^	-0.283^∗∗∗^	0.158^∗^	-0.579^∗∗∗^	-	
7. Anxiety symptoms^b^	-0.232^∗∗∗^	0.189^∗∗^	-0.163^∗∗∗^	0.253^∗∗∗^	-0.435^∗∗∗^	0.548^∗∗∗^	-


*^*a*^Change scores from T0 to T1. ^*b*^Change scores from T0 to T2. ^∗^*p* < 0.05. ^∗∗^*p* < 0.01. ^∗∗∗^*p* < 0.001.*

### Unique Indirect Effects of Mediators on Well-Being and Distress

Outcomes of the simple mediation models are shown in Table 4. All coefficients of the c-paths and a-paths were significant, indicating that CFT had a significant positive effect on all outcomes and mediators compared to the waitlist condition. Coefficients of the b-paths revealed that all four mediators were significantly associated with well-being and depressive symptoms, though the magnitude of the association differed across outcomes and mediators. For anxiety symptoms, a significant relationship was found with all mediators but positive affect (*p* = 0.061). For well-being, the 95% CIs of the indirect effects did not contain zero in any model, indicating that the effects of CFT versus waitlist on well-being were significantly mediated through T0-T1 changes in self-reassurance, self-criticism, positive affect and negative affect. With regard to psychological distress, the 95% CIs of the indirect effects revealed that the effects on both depressive and anxiety symptoms were significantly mediated by changes in self-reassurance and self-criticism. Also affect was found to play a mediating role in the effectiveness of the CFT intervention in improving psychological distress, whereby effects on depressive symptoms were found to be mediated by changes in positive affect and effects on anxiety symptoms were found to be mediated by changes in negative affect.

**Table 4:** Outcomes of simple mediation models assessing indirect effects of mediators on changes in well-being, depressive symptoms and anxiety symptoms compared to the waitlist control condition.

CFT vs. WLC	c-path^a^	a-path^a^	b-path^a^	Indirect effects	
				
				ab	95% CI
**MHC-SF – well-being**					
FSCRS – self-reassurance	0.346***	2.520***	0.040***	0.101	0.041, 0.182
FSCRS – self-criticism	0.347***	-2.860***	-0.015*	0.044	0.004, 0.106
PANAS – positive affect	0.352***	2.088**	0.025**	0.053	0.016, 0.115
PANAS – negative affect	0.346***	-2.063**	-0.018*	0.036	0.004, 0.091
**HADS – depressive symptoms**					
FSCRS – self-reassurance	-1.107**	2.372***	-0.179***	-0.424	-0.790, -0.170
FSCRS – self-criticism	-1.106**	-2.636***	0.096**	-0.253	-0.541, -0.069
PANAS – positive affect	-1.107**	2.023**	-0.145***	-0.294	-0.636, -0.091
PANAS – negative affect	-1.106**	-2.061**	0.082*	-0.169	-0.491, 0.002
**HADS – anxiety symptoms**					
FSCRS – self-reassurance	-1.367***	2.387***	-0.157**	-0.375	-0.685, -0.150
FSCRS – self-criticism	-1.369***	-2.659***	0.073*	-0.195	-0.489, -0.017
PANAS – positive affect	-1.356***	1.989**	-0.069	-0.137	-0.370, 0.010
PANAS – negative affect	-1.362***	-2.098**	0.125***	-0.263	-0.601, -0.062


*FSCRS, Forms of Self-Criticising/Attacking and Self-Reassuring Scale; HADS-A, Hospital Anxiety and Depression Scale – anxiety subscale; HADS-D, Hospital Anxiety and Depression Scale – depression subscale; PANAS, Positive and Negative Affect Schedule. ^*a*^Values are unstandardised betas. ^∗^*p* < 0.05. ^∗∗^*p* < 0.01. ^∗∗∗^*p* < 0.001.*

### Combined Indirect Effects of Mediators on Well-Being and Distress

**FIGURE 2:**
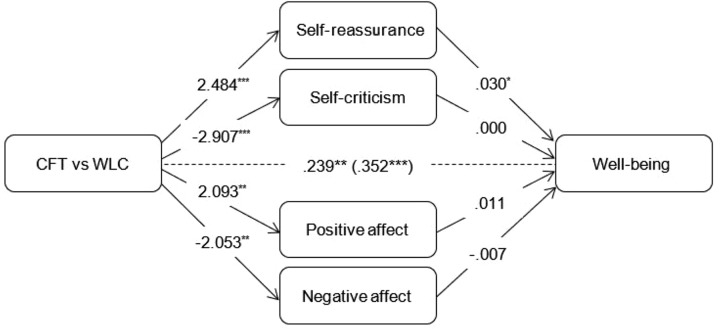
Outcomes of multiple mediation models assessing indirect effects of mediators on changes in well-being compared to the waitlist control condition. Total effect (c-path) is given in parentheses. *^∗^p <* 0.05. *^∗∗^p <* 0.01. ^∗∗^*p* < 0.001.

**FIGURE 3:**
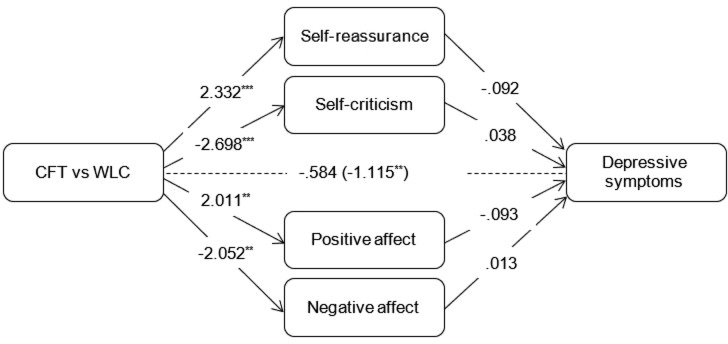
Outcomes of multiple mediation models assessing indirect effects of mediators on changes in depressive symptoms compared to the waitlist control condition. Total effect (c-path) is given in parentheses. ^∗∗^*p* < 0.01, *^∗∗∗^p <* 0.001.

**FIGURE 4:**
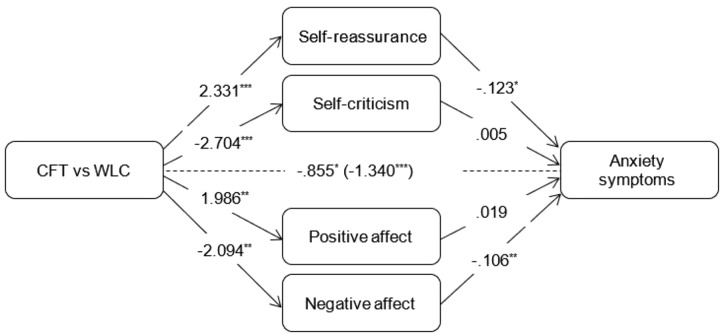
Outcomes of multiple mediation models assessing indirect effects of mediators on changes in anxiety symptoms compared to the waitlist control condition. Total effect (c-path) is given in parentheses. *^∗∗^p <* 0.01*.^∗∗∗^p <* 0.001.

For each outcome, additional mediational analyses were conducted wherein all mediators were simultaneously added to the model. Coefficients of the c-paths and a-paths were similar to those in the simple mediation models. As for the simple models, coefficients of the b-paths were similar for well-being but considerably smaller for depressive and anxiety symptoms (see Figures 2–4). In a combined mediation model, only T0-T1 changes in self-reassurance remained a significant mediator of the intervention effect on well-being (ab = 0.075, 95% CI [0.003, 0.163]). For depressive symptoms, only changes in positive affect were found to account for the effectiveness of the intervention (ab = -0.188, 95% CI [-0.503, -0.001]). Changes in self-reassurance (ab = -0.287, 95% CI [-0.658, -0.005]) and negative affect (ab = -0.222, 95% CI [-0.555, -0.035]) simultaneously mediated the effect of CFT relative to the waitlist condition on anxiety symptoms.

## Discussion

The present study was the first to examine the mediating role of self-reassurance, self-criticism and positive/negative affect in explaining the effectiveness of CFT, using data from a waitlisted RCT investigating the effectiveness of a guided self-help CFT intervention in an adult community sample with low to moderate levels of well-being and mild distress (Sommers-Spijkerman et al., 2018a). In doing so, this study sought to gain empirical support for four theoretical mechanisms of change underlying CFT. Previous findings from the RCT demonstrated that CFT participants significantly improved in terms of well-being as well as several domains of psychological distress, including depressive and anxiety symptoms, compared to the waitlist control condition. Additionally, significant pre-post improvements were observed in self-reassurance, self-criticism, positive affect and negative affect, which relate to four major therapeutic change processes outlined in the CFT model (Sommers-Spijkerman et al., 2018a). Those findings were corroborated in the current study.

In the simple mediation models, changes in well-being and psychological distress in the CFT group versus the waitlist group were significantly mediated by improvements in self-reassurance and self-criticism. Additionally, changes in positive affect were found a significant mediator of the intervention effect on well-being and depressive symptoms while changes in negative affect were found a significant mediator of the intervention effect on well-being and anxiety symptoms. The combined mediation models revealed that the effects of the CFT intervention on well-being were mediated merely through improvements in self-reassurance. With regard to psychological distress, the impact of CFT relative to the waitlist condition on anxiety symptoms was found to be simultaneously mediated by pre-post changes in self-reassurance and negative affect, while changes in positive affect emerged as the sole significant mediator of the intervention effect on depressive symptoms. Thus, it seems that all four variables – self-reassurance, self-criticism and positive/negative affect – mediated the effectiveness of the CFT intervention, but which mechanism mattered most varied per outcome.

A number of findings are worth highlighting. One is that self-reassurance appears a primary mechanism through which CFT operates when it comes to two out of three outcomes (i.e., well-being and anxiety symptoms), thereby lending support for the theoretical framework of CFT. According to Gilbert (2014), the CFT approach comprises several major steps, including: (1) cultivating and building compassionate attributes and skills conducive to the experience of a compassionate self; (2) developing a compassionate sense of self using a range of exercises and techniques including imagery, breathing and voice tones; and (3) using one’s compassionate self to engage with and tackle specific problems, such as self-criticism, shame or depressive symptoms. The cultivation of self-reassurance is assumed crucial to the change process during CFT, which is reflected by the first two steps.

Nonetheless, our analyses with depressive symptoms do not corroborate the notion that the cultivation of the ability to reassure oneself in the face of setbacks is the primary and central working mechanism in CFT. A combined mediation model indicated that effects of the CFT self-help intervention on depressive symptoms were solely mediated by changes in positive affect. A caveat here is that pre-post changes in positive affect and self-reassurance strongly correlated with one another (*r* = 0.52). Unfortunately, our data are inconclusive regarding the direction of this relationship. Although the theoretical model underlying CFT presumes that changes in (the processing of) positive affect evolve from the cultivation of self-reassurance, this relationship might work the other way around as well. Drawing on the broaden-and-build theory (Fredrickson, 1998), it is not unlikely that the experience of positive affect encourages people to build compassionate skills. Future research may provide more clarity as to how these change processes relate to one another.

In support of our finding that positive affect has a mediating impact on the effects of CFT on depressive symptoms, multiple studies have demonstrated that psychological interventions fostering positive affect are effective in treating depression (Santos et al., 2013). Nonetheless, this finding is contradictory with a recent experimental study which concluded that compassion-focused interventions may reduce depressive symptoms especially by improving individuals’ ability to tolerate negative affect (Diedrich et al., 2016). In this regard it should be noted, however, that the study by Diedrich et al. (2016) was conducted in a clinically depressed sample. It is very well possible that the working mechanisms of CFT function differently in clinical populations versus subclinical populations such as our sample. For instance, while it may be possible to address all four mechanisms simultaneously in a subclinical sample with only mild psychological symptoms, in a clinical sample with more severe levels of distress, it can be expected that self-criticism and negative affect must be mitigated first as to make “space” for developing self-reassurance and positive affect. In more severely distressed samples, yet also in non-clinical samples such as in our study, it may be crucial as well to address fears, blocks and resistances to compassion which is also a core part of CFT (Gilbert, 2009). Previous work suggests that fear of self-compassion and of receiving compassion from others may impede experiences of warmth, soothing and reassurance and fuel depressive and anxiety symptoms (Gilbert et al., 2011, 2014; Hermanto et al., 2016; Matos et al., 2017c; Trindade et al., 2018).

Strikingly, self-criticism came out as the least important working mechanism. In the combined models, neither changes in well-being nor psychological distress were found to be mediated by improvements in self-criticism, even though self-critical thinking was also rather actively targeted during the intervention. One possible explanation is that an increase in self-reassurance is a prerequisite to effective coping with self-criticism. As mirrored in step 3 above, self-reassurance is thought to play a key role in activating secondary working mechanisms, including self-criticism. Following the theoretical underpinnings of CFT, participants are likely to adopt a more compassionate or reassuring sense of self as they learn the attributes and skills of compassion, which, in turn, enables them to step out of (habitual) self-critical patterns of thinking and to respond to unpleasant or feared emotions with sympathy, empathy and non-judgement (Gilbert, 2014). Another possible explanation is that participants adopt skills for reducing feelings of personal inadequacy over a longer time interval than skills for a compassionate mind. This is consistent with earlier reported findings from the RCT by Sommers-Spijkerman et al. (2018a) indicating that self-reassurance appeared rather stable once learned (at post-intervention) while self-criticism scores, as measured with the inadequate self subscale of the FSCRS, further improved between post-test and 3-month follow-up. Finally, this finding may be accounted for by the use of a subclinical sample with relatively low baseline levels of self-criticism. Self-criticism might play a more vital role in clinical samples.

Single mediation analyses revealed that affect played a mediating role when examining the effects of CFT on well-being, as was the case when examining the effects on depressive and anxiety symptoms. In contrast to this finding, the combined models showed mediation by improvements in positive or negative affect when examining the effects on psychological distress but not when examining the effects on well-being, suggesting that changes in affect might constitute a major mechanism through which CFT helps reduce depressive and anxiety symptoms yet a second-order mechanism in the context of well-being. This inconsistency across outcome variables may be partly explained by the use of the PANAS to measure changes in affect. Whereas this self-report questionnaire measures only high arousal (positive and negative) emotions, it is presumed that improvements in well-being during CFT are mainly achieved through fostering low arousal positive affective states such as safeness, calmness and contentment. Due to the constraints of the measurement instrument, we cannot rule out that relevant changes in positive affective states underlying well-being that had been expected to occur among the participants could not have been detected. It is possible that the PANAS was better able to detect affective changes linked to improvements in psychological distress, notably anxiety. In this respect, it should be noted, however, that low and high arousal positive emotions are likely to cluster with one another. For instance, a study of Fredrickson et al. (2008) demonstrated that practising loving-kindness meditation (LKM), another compassion-based intervention, leads to improvements in both low arousal (e.g., contentment, love, gratitude) and high arousal positive affect (e.g., amusement, joy, pride). In this light, we believe it is reasonable to expect that CFT, like LKM, facilitates low arousal positive affect as well.

Based on the findings of the current study, it seems tenable that the working mechanisms of CFT may be dependent upon the intended outcome of therapy (i.e., increase in well-being or decrease in psychological distress). When the focus is on improving well-being, adopting a soothing and reassured sense of self is likely to matter most. In this case, strengthening the soothing and affiliation system, characterised by positive affective states of calmness, safeness and contentment, is at the core of CFT. However, when CFT is aimed at relieving psychological distress, it becomes equally or perhaps even more important to pay attention to negative affective states characteristic of the threat protection system such as anxiety, guilt or shame. Then, it seems especially important that participants learn to apply the acquired compassionate attributes and skills to regulate their affective states and process emotions in a more adaptive manner.

### Limitations

The current study has several limitations. Firstly, compassion was not measured in all its different flows. We focused on self-compassion, and more specifically self-reassurance, for two reasons: (1) the RCT employed an intervention which is primarily focused on cultivating compassion for the self, and (2) at the time the RCT was designed there were no psychometrically valid and reliable measures available that examined compassion for and from others. Secondly, our data allowed for examining a small selection of possible mediating variables. Thirdly, as mediators were measured at baseline and post-test, but not during the intervention, we cannot establish whether the relationship between mediators and outcomes is causal in nature (Murphy et al., 2009). Fourthly, it is likely that the intervention was not identical in every case, for several reasons. CFT was implemented in a flexible manner, adjusting to the circumstances, needs, preferences and progress of the participants. For instance, not every participant performed exactly the same exercises or performed the exercises at the same frequency or in the same order. Furthermore, the email support was provided by five different counsellors who may have used slightly different approaches. Due to these differences, not every mediator may have been targeted to the same extent across participants. To minimise the influence of the counsellor on intervention effects, participants were randomly assigned to a counsellor, all counsellors received the same instructions and counsellors participated in weekly supervision meetings. Fifthly, since the waitlist group received the intervention after the 3-month follow-up, we were not able to explore mediators of changes at 9-month follow-up.

### Future Directions

Compassion-focused therapy is increasingly implemented as therapeutic tool in clinical practise even though it remains as yet unclear how exactly it works. Although the current study provides preliminary evidence for four possible working mechanisms underlying the effectiveness of CFT, additional work is needed to replicate and build on these initial findings. To be able to further refine current CFT programmes, optimise its effectiveness and tailor it for various target groups with differing characteristics, a vital step is to investigate the presumed mechanisms of change in more detail. Studies designed to measure both mediators and outcomes at multiple intervals over the course of the intervention are recommended to shed more light on the existence and course of therapeutic change processes as well as their interplay (Kraemer et al., 2002). Future research also needs to test the proposition that changes in self-reassurance occur first and subsequently facilitate changes in self-criticism and affect. However, future research should not be limited to achieving a deeper understanding of the mediating role of self-reassurance, self-criticism and positive/negative affect. Self-reassurance is only one facet of (self-)compassion. In order to provide a more complete picture of the mediating role of compassion, other facets of self-compassion as well as compassion to and from others should also be taken into account. From a practitioner’s point of view, it would be worthwhile to elucidate which facets of compassion are likely to change by which parts of the intervention (e.g., by which types of exercises). Suitable methods for revealing the active ingredients of CFT can be found in, among others, experience sampling studies and dismantling studies. Alongside cultivating compassion, a main goal in CFT is to address fears of (receiving) compassion (Gilbert, 2014). This would be an important mediator to consider in future research, particularly in the context of growing evidence for the crucial role of fears of compassion in CFT and its impact on treatment outcome. As shown by Hermanto et al. (2016), the relationship between self-criticism and depression is moderated by fear of receiving compassion whereby high fear of compassion increases the depressogenic effect of self-criticism and vice versa for low fear of compassion. With regard to affect regulation as mechanism of change, physiological processes may also be of interest. Previous work has shown that practising compassion has a favourable impact on heart rate variability (Rockliff et al., 2008; Kirby et al., 2017a; Matos et al., 2017c) through increasing parasympathetic activity (Stellar et al., 2015) which is characteristic of the soothing and affiliation system. Moreover, CFT may build positive psychological resources such as hope, optimism and gratitude (Neff et al., 2007; Petrocchi and Couyoumdjian, 2015; Yang et al., 2016) and impact many other (transdiagnostic) processes underlying mental health including shame, rumination, experiential avoidance and psychological inflexibility (e.g., Boersma et al., 2014). These and other relevant processes also deserve attention as possible mechanisms of change in CFT. Finally, the current study investigated mediators of changes in CFT in a non-clinical population. Future work may reveal whether improvements in well-being and distress during CFT follow similar or different trajectories in both non-clinical and clinical populations.

## Conclusion

The present study provides preliminary empirical evidence that CFT operates through multiple mechanisms of change, namely through cultivating self-reassurance, reducing self-criticism and regulating positive and negative affect. Which of these mechanisms lies at the heart of the therapeutic change process seems to depend on the goal of CFT, i.e., improving well-being or relieving psychological distress. To further advance the development of CFT, there is a need for more research in this area applying more rigorous designs, such as dismantling studies, to replicate these findings, to further explore the interrelationships between relevant therapeutic change processes and to elucidate other active ingredients in CFT.

## Author Contributions

MS-S and HT designed the study. MS-S conducted the statistical analyses and wrote the manuscript. HT, KS, and EB were involved in drafting the manuscript and provided the critical review. All authors read and approved the manuscript.

## Conflict of Interest Statement

The authors declare that the research was conducted in the absence of any commercial or financial relationships that could be construed as a potential conflict of interest.
